# Comparative Genomics Analysis of Keratin-Degrading *Chryseobacterium* Species Reveals Their Keratinolytic Potential for Secondary Metabolite Production

**DOI:** 10.3390/microorganisms9051042

**Published:** 2021-05-12

**Authors:** Dingrong Kang, Saeed Shoaie, Samuel Jacquiod, Søren J. Sørensen, Rodrigo Ledesma-Amaro

**Affiliations:** 1 Section of Microbiology, Department of Biology, University of Copenhagen, 2100 Copenhagen, Denmark; sjs@bio.ku.dk; 2 Imperial College Centre for Synthetic Biology, Imperial College London, London SW7 2AZ, UK; 3 Department of Bioengineering, Imperial College London, London SW7 2AZ, UK; 4 Centre for Host-Microbiome Interactions, Faculty of Dentistry, Oral & Craniofacial Sciences, King’s College London, Lodon SE1 9RT, UK; saeed.shoaie@kcl.ac.uk; 5 TERRA Research and Teaching Centre, Microbial Processes and Interactions (MiPI), Gembloux Agro-Bio Tech, University of Liège, 5030 Gembloux, Belgium; 6 Science for Life Laboratory, Department of Protein Science, KTH Royal Institute of Technology, 114 17 Stockholm, Sweden; 7 Agroécologie, AgroSup Dijon, INRAE, Université de Bourgogne Franche-Comté, F-21000 Dijon, France; samjqd@gmail.com

**Keywords:** keratinous materials, metabolic potential, genome mining, gene clusters, degradation pathways

## Abstract

A promising keratin-degrading strain from the genus *Chryseobacterium* (*Chryseobacterium* sp. KMC2) was investigated using comparative genomic tools against three publicly available reference genomes to reveal the keratinolytic potential for biosynthesis of valuable secondary metabolites. Genomic features and metabolic potential of four species were compared, showing genomic differences but similar functional categories. Eleven different secondary metabolite gene clusters of interest were mined from the four genomes successfully, including five common ones shared across all genomes. Among the common metabolites, we identified gene clusters involved in biosynthesis of flexirubin-type pigment, microviridin, and siderophore, showing remarkable conservation across the four genomes. Unique secondary metabolite gene clusters were also discovered, for example, ladderane from *Chryseobacterium* sp. KMC2. Additionally, this study provides a more comprehensive understanding of the potential metabolic pathways of keratin utilization in *Chryseobacterium* sp. KMC2, with the involvement of amino acid metabolism, TCA cycle, glycolysis/gluconeogenesis, propanoate metabolism, and sulfate reduction. This work uncovers the biosynthesis of secondary metabolite gene clusters from four keratinolytic *Chryseobacterium* species and shades lights on the keratinolytic potential of *Chryseobacterium* sp. KMC2 from a genome-mining perspective, can provide alternatives to valorize keratinous materials into high-value bioactive natural products.

## 1. Introduction

Keratin is the most abundant protein in epithelial cells, constituting the bulk of epidermal appendages such as hair and feather [1,2]. Keratinous materials represent an abundant protein source, particularly originating from the commercial slaughterhouses or poultry farms [3]. They contain peptides and amino acids, which are renewable natural resources with great potential in sustainable development [4]. However, keratin is an insoluble protein with highly cross-linked disulfide bonds giving it a tough and recalcitrant structure [5]. Many attempts have been made to hydrolyze keratinous materials in terms of physicochemical treatment, enzymatic hydrolysis, and microbial conversion [6,7]. The hydrolysis products of keratinous materials have been used for animal feed [8] and fertilizer [9] based on conventional processing.

Microorganisms represent one of the most important sources of bioactive natural products, which have the potential to generate compounds such as antibiotics, biofuels, and natural pigments derived from cellular metabolites [10,11]. For example, *Yarrowia lipolytica* has been used to convert different renewable feedstocks to high-value metabolites [12]. Similarly, *Escherichia coli* has become one of the best cell reactors to produce alcohols, organic acids, biodiesel, and even hydrogen by utilizing renewable resources [13]. Other bacteria such as *Bacillus subtilis* [14], *Caldicellulosiruptor bescii* [15], *Corynebacterium glutamicum* [16], and *Ruminococcaceae* [17] were identified and evaluated with the capacity to generate different products by converting renewable carbon sources. Notably, some microorganisms were reported to degrade keratinous waste effectively [18]. Exploring keratinolytic potential of these microbes to generate high-value-added products is an important step to recycle and valorize keratinous materials.

Molecular mechanisms of microbial keratin degradation are still not fully understood, while genome sequencing offers possibilities to reveal the metabolic potential behind efficient microbial degradation [19]. Novel keratinolytic enzymes were identified from the genome of *Bacillus pumilus* 8A6, an efficient keratin degrader [20]. Furthermore, going beyond the degradation reaction itself, genomes can also be minded for valuable accessory functions of interest, adding more values to the microbial conversion processes. For instance, gene clusters and biosynthesis pathways of secondary metabolites could be disclosed from genomes via adequate analysis tools [21,22]. A total of 104 putative biosynthetic gene clusters for secondary metabolites were predicted from nine *Ktedonobacteria* genomes [23]. Secondary metabolites were identified and linked to gene clusters based on the comparison and mining of six genomes belonging to diverse *Aspergillus* species, successfully fueling industrial biotechnology initiatives and medical research [24]. Therefore, using the genomes of keratinolytic microbial species in a similar way would represent a promising approach to discover biosynthetic gene clusters of secondary metabolites of interest, excavating the full application potential of these microbes.

Recently, several studies based on different environments have revealed the remarkable potential of representative taxa from the *Chryseobacterium* genus for keratin degradation using isolation, activity tests and genome sequencing [25,26]. A novel strain *Chryseobacterium* sp. KMC2 with high keratin degradation capacity was obtained from our enriched keratinolytic microbial consortium, which was identified base on the 16S rRNA gene sequencing [27]. In this study, the genome of *Chryseobacterium* sp. KMC2 was sequenced and compared with publicly available genomes of other keratinolytic *Chryseobacterium* species to clarify the genomic basis of keratin degradation, and to unravel hidden biosynthetic gene clusters of interest. Subsequently, the metabolic pathways associated with keratin degradation were constructed, providing deeper insight into the yet obscure keratinolytic processes. This work reveals the keratinolytic potential of *Chryseobacterium* species and mined potential accessory gene clusters of secondary metabolites, which could i) contribute to optimizing the processes of keratin degradation and ii) broaden the perspective to generate added-value products from keratin hydrolysate.

## 2. Materials and Methods

### 2.1. DNA Preparation

*Chryseobacterium* sp. KMC2 was isolated and identified from a keratinolytic microbial consortium enriched from a soil sample [19,27,28]. The keratinolytic capacity of *Chryseobacterium* sp. KMC2 was confirmed, and the role of this strain in the consortium was evaluated to be crucial to achieve efficient keratin degradation [27]. *Chryseobacterium* sp. KMC2 was inoculated to LB medium, and cultured overnight (200 rpm, 30 °C). Two milliliters of the suspension were centrifuged and collected to prepare the DNA extraction, performed by using by FAST Soil DNA Kit (MP Biomedicals, Solon, OH, USA) according to the manufacturer’s instructions.

### 2.2. Genome Sequencing, Assembling, and Functional Annotation

The genome sequencing was performed by an Illumina Miseq instrument (2 × 250 bp paired-end reads) (Illumina, San Diego, CA, USA) at the University of Copenhagen by using TruSeq DNA Library Preparation Kits v2 (Illumina, San Diego, CA, USA), according to the manufacturer’s instructions. Raw reads were treated and assembled to contigs on CLC Genomic Workbench 8.5.1. The obtained contigs were validated using QUAST 4.5 [29]. Genes were predicted from the contigs and further annotated with Prokka v1.14.5 [30]. Predicted genes were submitted to eggNOG 5.0 database to obtain an integrated functional annotation and classification [31].

### 2.3. Whole-Genome Phylogenetic Analysis

To determine the phylogenetic origin of *Chryseobacterium* sp. KMC2 in the *Chryseobacterium* genus, the whole-genome sequences of 11 publicly available *Chryseobacterium* species were downloaded from NCBI database to construct a phylogenetic tree. The whole-genome sequence-based phylogenetic tree was inferred by using an online pipeline: The Reference sequence Alignment based Phylogeny builder (REALPHY 1.12) [32], based on the merge reference alignments. All genomes were merged to generate the reference sequences. Sequence reads from each query genome were chopped into 50 bp fragments and mapped to the reference via Bowtie 2 [33]. Multiple sequence alignments were reconstructed, and a phylogenetic tree was inferred by PhyML 3.0 [34]. Visualization of the obtained phylogenetic tree was generated by iTOL v5 [35]. Moreover, gene presence–absence patterns among these genomes were analyzed with M1CR0B1AL1Z3R server [36], which was further used to calculate homology using GLOOME with fixed gene gain/loss ratio [37]. The phylogenetic tree was visualized by FigTree v1.4.4 (tree.bio.ed.ac.uk/software/figtree/) (accessed on 15 April 2021), using the branches to display the gene gain and loss rates. Average Nucleotide Identity (ANI) was calculated using OrthoANI [38].

### 2.4. Secondary Metabolite Gene Cluster Detection and Annotation

Assembled contigs of four *Chryseobacterium* species were uploaded to antiSMASH 5.0 secondary metabolite genome mining web platform [21]. Predicted secondary metabolites gene clusters from *Chryseobacterium* sp. KMC2 were compared with other keratinolytic *Chryseobacterium* species. Gene annotation of each cluster from *Chryseobacterium* sp. KMC2 was performed by Prokka v1.14.5 [30] and BLASTP with the NCBI database. The best match sequencing ID was recorded for the annotated genes. Synteny and features of conservative secondary metabolite gene clusters were analyzed by using Easyfig 2.2.2, showing the similarity of gene sequences [39]. Feature comparison of amino acid sequences and motifs from core synthetic genes were analyzed by using Clustal Omega [40] to get the multiple sequence alignment and using Seq2logo to generate sequence logo [41].

### 2.5. Metabolic Networks Construction and Protease Families Prediction

The genomes of *Chryseobacterium* sp. KMC2 and other three *Chryseobacterium* species were submitted to GhostKOALA [42] to obtain the KO number for each gene, then genes were assigned to different metabolic pathways and functional categories. Following the metabolic networks construction of *Chryseobacterium* sp. KMC2 was achieved through mapping the annotated enzyme genes to KEGG [43] reference pathway and Biocyc database [44] manually. Protease families’ prediction was performed based on CDSs sequence alignment against the MEROPS peptidase database according to the peptide-based functional annotation principle of Peptide Pattern Recognition (PPR) [45] implemented by the Homology to Peptide Pattern method [46]. The signal peptides of putative proteases were predicted by the SignalP 5.0 Server [47].

## 3. Results and Discussion

### 3.1. Genome Feature Comparison of Four Keratinolytic Chryseobacterium Species

*Chryseobacterium* sp. KMC2 originated from a river-bank soil sample, and displayed a potent degradation ability toward milled pig bristle and hooves [27,28]. The genome of *Chryseobacterium* sp. KMC2 was sequenced and compared to three reference genomes of *Chryseobacterium* species (Table 1).

**Figure 1:**
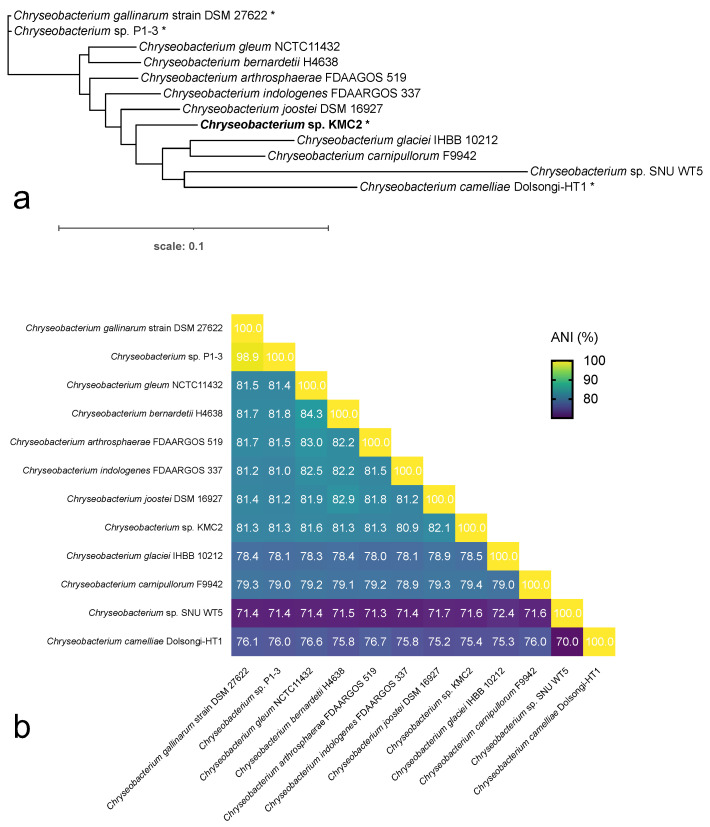
Analysis of *Chryseobacterium* genomes. (**a**) The whole-genome sequence-based phylogenetic tree of *Chryseobacterium* species, based on the merge reference alignments of all genomes. Branch length represents divergence, and stars show the keratinolytic *Chryseobacterium* species. (**b**) Overall orthologous average nucleotide identity (ANI) among pairwise *Chryseobacterium* genomes. Values in heatmap indicate the similarity percentage.

Including: (i) *Chryseobacterium camelliae* Dolsongi-HT1, isolated from green tea leaves [48]; (ii) *Chryseobacterium gallinarum* strain DSM 27622, isolated from chicken [49]; and (iii) *Chryseobacterium* sp. P1-3 isolated from poultry waste [50], which all display keratinolytic capacity. *Chryseobacterium* sp. KMC2 showed distinct genome feature from the other known keratinolytic strains. The genome size of *Chryseobacterium* sp. KMC2 is 5.28 Mbp, larger than the other three genomes, which ranged from 4.38 Mbp to 4.63 Mbp. A total of 4773 genes were predicted from *Chryseobacterium* sp. KMC2 genome, and more than 4000 genes were predicted from the other three genomes. Besides, the GC content ranges from 36.33% to 41.80% in *Chryseobacterium* species genomes. Furthermore, the whole-genome phylogenetic tree was constructed with other eight publically available *Chryseobacterium* species genomes (Figure 1a), showing the close phylogenetic relationship between *Chryseobacterium gallinarum* strain DSM 27622 and *Chryseobacterium* sp. P1-3. Notably, *Chryseobacterium* sp. KMC2 and *Chryseobacterium camelliae* Dolsongi-HT1 have closer homology with other *Chryseobacterium* species. Average Nucleotide Identity (ANI) among *Chryseobacterium* genomes was calculated (Figure 1b), which shows that the similarity percentages among most pairwise genome are around 70% to 85%. While the genomes of *Chryseobacterium gallinarum* strain DSM 27622 and *Chryseobacterium* sp. P1-3 display highly similarity (98.9%). On the other hand, the genome-wide relationship was also evaluated by gene loss and gain dynamics (Supplementary Figure S1). The genomes of the four keratinolytic *Chryseobacterium* species were not present in the same branch of the phylogenetic trees. This result suggests that the keratinolytic capacity is a generalist trait that occurs in several places without links to phylogeny.

### 3.2. Metabolic Potential Comparison of Four Keratinolytic Chryseobacterium Genomes

**Figure 2:**
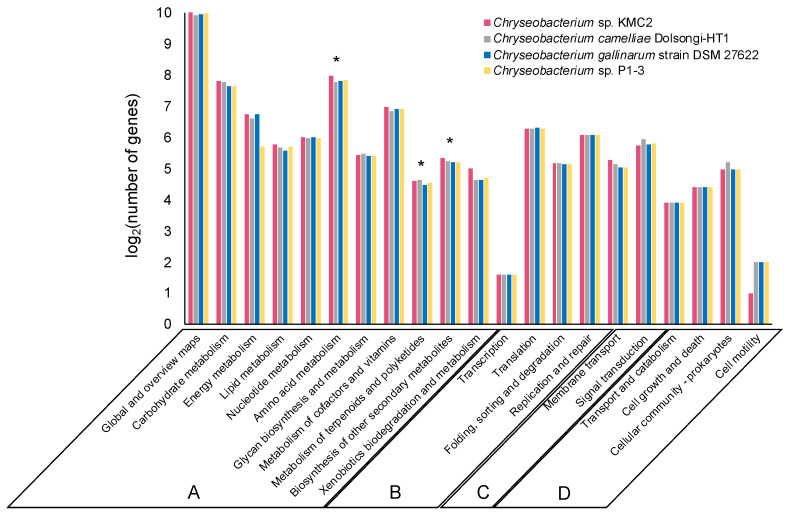
Comparison of KEGG function classification amongst four *Chryseobacterium* genomes. Functional categories: Metabolism (**A**), Genetic information processing (**B**), Environmental information processing (**C**), and Cellular processes (**D**). The stars show the sub-categories: Amino acid metabolism, metabolism of terpenoids and polyketides, and biosynthesis of other secondary metabolites.

About 40% of the genes from the four genomes were annotated and classified into various functional categories based on the KEGG database. The vast majority of annotated genes belonged to metabolism, genetic information processing, environmental information processing, and cellular processes (Figure 2). The functional categories of the genomes were overall highly similar, with ~85% of annotated genes assigned to “metabolism” (category A) which included ~1.000 genes into the sub-category “global and overview maps”. Additionally, about 8% and 4% annotated genes from each genome were assigned to “genetic information processing” (category B) and “environmental information processing” (category C), respectively. The remaining annotated genes belonged to “cellular processes” (category D), which occupied 3% of the annotated genomes approximately.

Remarkably, each genome had more than 200 genes assigned into the “amino acid metabolism” sub-category. Keratin is mainly composed of amino acids [3], which is ultimately the operational nutrient source exploited for microbial growth. Numerous amino acid metabolism-related enzymes were annotated, revealing the genetic potential of these *Chryseobacterium* strains for using keratin materials as carbon source.

Of particular interest, several biosynthesis genes of secondary metabolites were detected from the genomes, of which more than 20 genes were assigned to “metabolism of terpenoids and polyketides” and around 40 genes were assigned to “biosynthesis of other secondary metabolites” sub-category (Figure 2). Terpenoids are a group of natural products with diverse commercial applications, which have been produced from microbial cell factories [51]. Many polyketides are considered as significant natural products with broad applications in the agriculture and pharmaceutical industry [52]. The metabolic pathways related to polyketides biosynthesis are well understood in some microorganisms like *Streptomyces* which play a crucial role in industrial bioproduction [53]. This result indicates that these *Chryseobacterium* strains could have the potential to synthesize high-value secondary metabolites such as terpenoids and polyketides from keratinous materials.

### 3.3. Mining and Comparing Secondary Metabolite Gene Clusters

**Figure 3:**
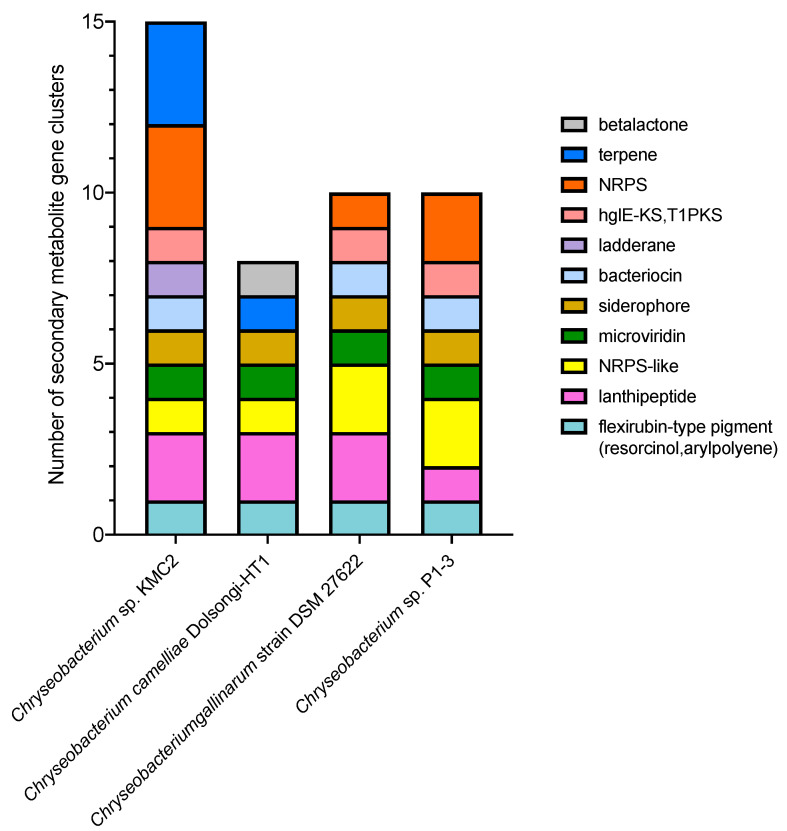
Composition of secondary metabolite gene clusters from four *Chryseobacterium* genomes.

Genome mining is an effective approach to discover new bioactive natural products from microorganisms based on “signature genes” detection or searching for specific patterns in gene sequences [54]. To explore the potential of producing high value chemicals from these four *Chryseobacterium*, secondary metabolite gene clusters were predicted by using antiSMASH 5.0 mining pipeline (Figure 3). In total, eleven different secondary metabolite gene clusters were identified. *Chryseobacterium* sp. KMC2 possesses the largest number (15), while *Chryseobacterium camelliae* Dolsongi-HT1 has the fewest (8). Ten gene clusters were predicted from the other two strains. Five gene clusters are present in the four genomes, which are flexirubin-type pigment (resorcinol and arylpolyene), microviridin, lanthipeptide, NRPS-like, and siderophore. Remarkably, the flexirubin-type pigment is a typical metabolite produced from *Flavobacterium* [55]. Several species from *Chryseobacterium* were previously designated and known as *Flavobacterium* owing to similar characteristics with the yellow pigments [56]. Flexirubin-type pigment was isolated and characterized from *Chryseobacterium* sp. UTM-3T [57]. In addition, *Chryseobacterium* sp. KMC2 owns a unique gene cluster to produce ladderane. Another unique natural product is beta-lactone from *Chryseobacterium camelliae* Dolsongi-HT1. Ladderanes are hydrocarbon chains which were regarded as membrane lipid components produced by anammox (anaerobic ammonia-oxidizing) bacteria uniquely, but the production is not affordable due to their extremely low growth [58,59]. Secondary metabolite gene clusters of other eight *Chryseobacterium* genomes were also predicted, consisting of 14 different candidates (Supplementary Figure S2). These results demonstrate that various secondary metabolite gene clusters including both expected and unusual were discovered from *Chryseobacterium* genomes, which could turn into novel bioactive natural product sources.

### 3.4. Synteny Analysis and Features of Secondary Metabolite Gene Clusters

**Figure 4:**
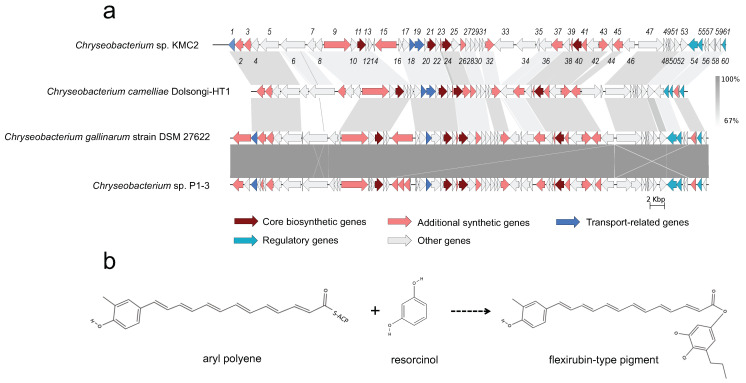
Flexirubin-type pigment gene cluster from four *Chryseobacterium* genomes. (**a**) Synteny analysis and features of flexirubin-type pigment gene cluster in *Chryseobacterium* species genomes. (**b**) The proposed biosynthetic reaction of flexirubin-type pigment. The detailed description of each gene can be found in Supplementary Table S1.

**Figure 5:**
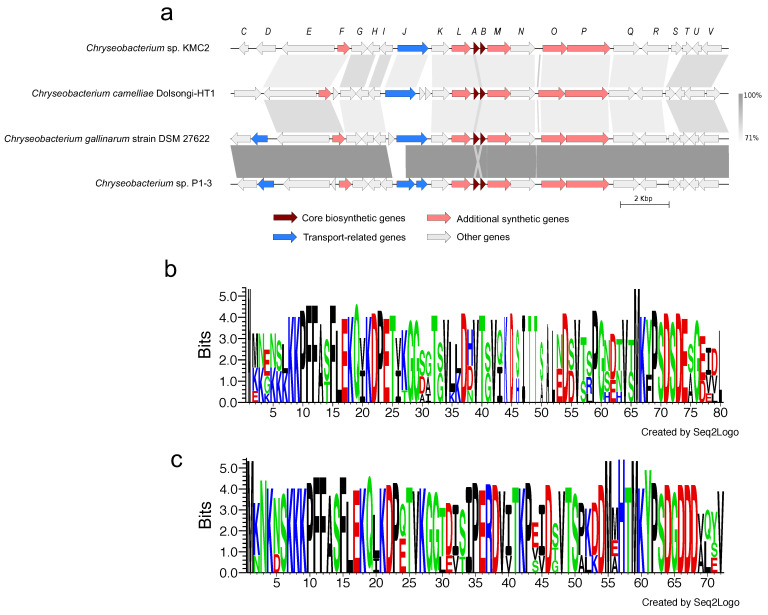
Microviridin gene cluster from four *Chryseobacterium* genomes. (**a**) Synteny analysis and features of microviridin gene cluster in *Chryseobacterium* genomes. (**b**) Amino acid sequence comparison of mdnA. (**c**) Amino acid sequence comparison of mdnB. The detailed description of each gene can be found in Supplementary Table S2.

**Figure 6:**
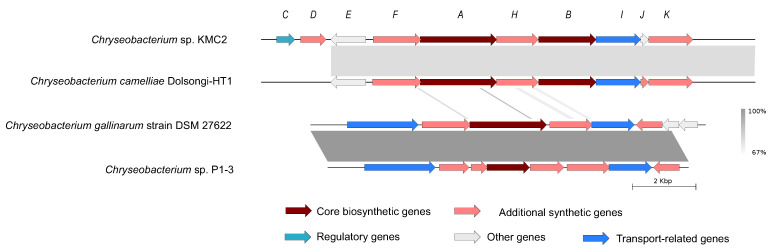
Siderophore gene cluster from four *Chryseobacterium* genomes. The detailed description of each gene can be found in Table S3.

Comparative genomics can reveal unique cluster and distribution patterns of secondary metabolites within species [60]. Five secondary metabolite gene clusters, predicted to be present in the four genomes, were selected to explore the homology among these *Chryseobacterium* strains. Three of them including flexirubin-type pigment, microviridin, and siderophore display a conserved gene cluster structure from synteny analysis (Figure 4, Figure 5 and Figure 6), while the other two showed no evident synteny relation (Supplementary Figures S3 and S4).

#### 3.4.1. Flexirubin-Type Pigment

Natural pigments have increasing applications in food, pharmaceutical, and textile industries, owing to their advantages such as non-toxic, biodegradable, and low allergenic potential compared to synthetic pigments [61]. In particular, flexirubin-type pigment has a potential antimicrobial and anti-tumoral activities [62]. Biosynthesis gene clusters of flexirubin-type pigment are conserved across the four tested genomes, especially within *Chryseobacterium gallinarum* strain DSM 27622 and *Chryseobacterium* sp. P1-3 (Figure 4a). A total of 61 biosynthesis-related genes of flexirubin-type pigment were predicted from *Chryseobacterium* sp. KMC2, including four core biosynthesis genes. One of the core biosynthesis genes was annotated as 3-oxoacyl-(acyl carrier protein) synthase III (*Flex11*), and the other three were annotated as Beta-ketoacyl synthases (*Flex21*, *Flex24*, and *Flex40*). Besides, transport-related genes and regulatory genes were predicted from the gene cluster. A previous study identified the molecular structure of flexirubin-type pigment isolated from *Chryseobacterium* sp. UTM-3T [57]. According to the products from core biosynthesis genes and their molecular structures, a proposition of biosynthesis pathway was established (Figure 4b), where flexirubin-type pigment is generated from resorcinol and arylpolyene. Further transcriptomics and metabolomics analysis would be required to confirm the validity of this potential pathway discovery.

#### 3.4.2. Microviridin

Microviridins represent a group of peptides under post-translational modifications, which have been mainly isolated from cyanobacteria and present potent serine-type protease inhibitory activities [63,64]. These properties could make microviridin serve as the natural antimicrobial agents for developing potential drugs. Biosynthesis gene clusters of microviridin from four *Chryseobacterium* genomes show a highly conserved structure with a similarity greater than 71% from most gene synteny analysis (Figure 5a). 22 biosynthesis genes of microviridin were predicted from *Chryseobacterium* sp. KMC2. Two core biosynthetic genes (A and B) were identified from genomes and transport-related genes were also been discovered. Besides, amino acid sequences of mvdA and mvdB were aligned, showing that multiple motifs from mvdA and mvdB are conserved (Figure 5bc). Interestingly, many keratinases were reported to be classified as serine proteases, acting on the molecular structure of keratin [65]. This suggests that microviridins may regulate keratinolytic activity. Further characterizing and manipulating the microviridin synthetic pathway could contribute to improving the keratin degradation efficiency.

#### 3.4.3. Siderophore

Siderophores are ferric ion-specific chelators to scavenge iron from the extracellular environment, which play important roles in virulence and oxidative stress tolerance in microorganisms [66]. It has been designed as a Trojan horse antibiotic to enter and kill pathogenic bacteria [67], and has been reported with the potential to decrease the growth of cancerous cells [68]. Biosynthesis gene cluster of siderophore shows a high synteny conservation among *Chryseobacterium* sp. KMC2 and *Chryseobacterium camelliae* Dolsongi-HT1, *Chryseobacterium gallinarum* strain DSM 27622 and *Chryseobacterium* sp. P1-3, respectively (Figure 6). A total of ten genes were predicted from siderophore biosynthesis cluster *of Chryseobacterium* sp. KMC2, and eight genes from the other three *Chryseobacterium* strains separately. Functional description of each gene related to siderophore biosynthesis in *Chryseobacterium* sp. KMC2 shows two core biosynthesis genes, and includes one regulatory gene and one transport-related gene. This further suggests that those siderophores are potentially fully functional molecular features that can be regulated on-demand and exported outside the cell when needed.

### 3.5. Metabolic Pathways of Keratin Utilization in Chryseobacterium sp. KMC2 Genome

**Figure 7:**
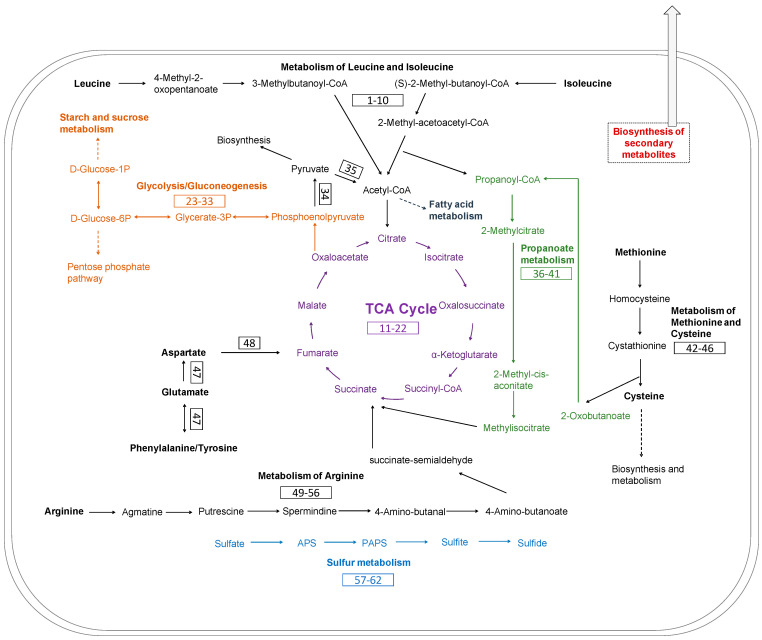
Metabolic pathways reconstruction of keratin utilization in *Chryseobacterium* sp. KMC2 genome. It includes amino acid metabolism, TCA cycle, glycolysis/gluconeogenesis propanoate metabolism, and sulfur metabolism. The number in the box represents the gene related to the metabolic pathway. The detailed description of each gene can be found in Table S7.

The main metabolic pathways related to keratin utilization in *Chryseobacterium* sp. KMC2 genome were investigated. These pathways included amino acid metabolism, TCA cycle, glycolysis/gluconeogenesis, propanoate metabolism, and sulfate reduction (Figure 7). A previous study suggested that abundant amino acids are released during microbial degradation and used as nutrient sources, such as leucine and aspartate [28]. The metabolic pathways of amino acid utilization were mapped from the genome of *Chryseobacterium* sp. KMC2. Most of the amino acids are converted into intermediates of the TCA cycle. For instance, arginine can be converted to succinate, then enter to TCA cycle after a multiple-steps enzyme reaction. Aspartate, tyrosine, phenylalanine, and glutamate could serve as the substrates to generate fumarate, thus being part of the TCA cycle. Besides, isoleucine turns into the substrates of 2-methyl-acetoacetyl-CoA after several enzymatic steps, which is then converted into acetyl-CoA and propanoyl-CoA via acetyl-CoA C-acyltransferase. Acetyl-CoA is an important intermediate, which can entry to the TCA cycle via citrate synthase [69]. It is also the precursors of fatty acid and polyketides biosynthesis [70]. Propanoyl-CoA serves as the critical substrate within propanoate metabolism and can also be used to make lipids [71,72]. On the other hand, methionine can be converted to 2-oxobutanoate, which is also an intermediate of propanoate metabolism. Subsequently, the methylmalonyl-CoA generated in propanoate metabolism enters into the TCA cycle via succinyl-CoA. Besides, the key enzymes of glycolysis/gluconeogenesis were found, indicating the potential to produce essential biomass components based on oxaloacetate from TCA cycle.

Evidence indicates that a source of redox is needed for complete keratin degradation with keratinases [73,74]. Several metabolites, including sulfite have been revealed to associate with efficient keratin degradation [75]. Moreover, sulfide is the metabolic product derived from the assimilatory sulfate reduction pathway, which not only increases the production of keratinase but also could participate in the breakdown of disulfide bonds [76]. The release of these metabolites during the microbial growth on keratinous materials probably leads to sulfitolysis [3,77]. Therefore, the complete metabolic pathway of assimilatory sulfate reduction was mapped in the genome, which could play important role during the keratin degradation process.

Furthermore, 286 proteases were predicted in *Chryseobacterium* sp. KMC2 genome, which are assigned to 61 protease families. 140 contain signal peptides, indicating that they are the potential secreted proteases (Supplementary Figure S5). Interestingly, about 78% secreted proteases from the prediction belong to serine proteases and metalloproteases, which is consistent with the fact that most known keratinases have been identified within these two protease families [65,77]. Following the development of sequencing technologies, increasing genomes of keratinolytic species have been unveiled, which provide a genomic perspective to reveal the molecular keratinolytic mechanisms. For instance, metabolic pathways related to keratin degradation such as enzymolysis and reduction of disulfide bonds were clarified through uncovering the genetic basis of microbial genomes [78]. The complex keratinolytic processes of *Streptomyces* sp. included protease secretion, iron uptake, spore formation, and resuscitation were recently revealed from a genome view [79]. Our results are in line with the notion that a redox environment is indeed required for efficient keratinolytic activity to occur. It is expected that the integrated metabolic pathways associated with keratinolytic processes will be deciphered along with more genomes sequencing and biochemical studies of relevant metabolic pathways.

## 4. Conclusions

In this work, the genomes from four *Chryseobacterium* species with keratinolytic ac-tivity were analyzed. Common and unique secondary metabolite gene clusters were mined from *Chryseobacterium* genomes, suggesting the potential to generate high value metabolites using keratin-rich wastes as the nutrient sources. Therefore, the use of these microorganisms could be an alternative way to valorize keratinous materials through microbial conversion. Furthermore, the metabolic pathways of keratin degradation from *Chryseobacterium* sp. KMC2 was studied from a genomic viewpoint. Nevertheless, there are still unknowns to link both metabolic pathways of keratinous utilization and the secondary metabolite biosynthesis. Understanding these connected pathways and their regulation will contribute to developing synthetic biology approaches to boost high value-added products from microbial keratin degradation.

## Figures and Tables

**Table 1:** Feature’s comparison of four *Chryseobacterium* species genomes.

Parameters	*Chryseobacterium* sp. KMC2	*Chryseobacterium camelliae* Dolsongi-HT1	*Chryseobacterium gallinarum* strain DSM 27622	*Chryseobacterium* sp. P1-3
Total length (bp)	5.276.159	4.376.354	4.633.632	4.628.764
Contigs	63	1	1	45
N50 (bp)	231.784	4.376.354	4.633.632	342.512
GC content (%)	36.33	41.80	37.30	37.02
Gene	4.773	4.012	4.161	4.906
CDS	4.692	4.009	4.151	4.939

## Data Availability

Reference genomes were downloaded from the NCBI database: *Chryseobacterium camelliae* Dolsongi-HT1 (GenBank: GCA_002770595.1), *Chryseobacterium gallinarum* strain DSM 27622 (GenBank: GCA_001021975.1), *Chryseobacterium* sp. P1-3 (GenBank: GCA_000738495.1), *Chryseobacterium gleum* NCTC11432 (GenBank: GCA_900636535.1), *Chryseobacterium bernardetii* H4638 (GenBank: GCA_003815955.1), *Chryseobacterium* *arthrosphaerae* FDAAGOS 519 (GenBank: GCA_003812705.1), *Chryseobacterium indologenes* FDAARGOS 337 (GenBank: GCA_002208925.2), *Chryseobacterium joostei* DSM 16927 (GenBank: GCA_003815775.1), *Chryseobacterium glaciei* IHBB 10212 (GenBank: GCA_001648155.1), *Chryseobacterium carnipullorum* F9942 (GenBank: GCA_003815855.1), and *Chryseobacterium* sp. SNU WT5 (GenBank: GCA_007362475.1). Raw sequencing data were deposited in the Sequence Read Archive (SRA) database under the BioProject number PRJNA686768 with an accession number SRR13278108. The assembled genome sequence of *Chryseobacterium* sp. KMC2 has been deposited at DDBJ/ENA/GenBank under the accession JAESIT000000000. Sequences of predicted proteases in *Chryseobacterium* sp. KMC2 genome are available in the figshare repository: doi.org/10.6084/m9.figshare.14453043.

## Supplementary Materials

The following are available online at https://www.mdpi.com/article/10.3390/microorganisms9051042/s1, Figure S1: Phylogenetic tree of 12 *Chryseobacterium* genomes based on gene presence–absence patterns. Figure S2: Composition of secondary metabolite gene clusters from eight *Chryseobacterium* genomes. Figure S3: Lanthipeptide gene cluster from four *Chryseobacterium* genomes. Figure S4: NRPS-like gene cluster from four *Chryseobacterium* genomes. Figure S5: Protease families predicted from *Chryseobacterium* sp. KMC2 genome. Table S1: The description of flexirubin-type pigment biosynthesis related gene cluster from *Chryseobacterium* sp. KMC2. Table S2: The description of microviridin biosynthesis related gene cluster from *Chryseobacterium* sp. KMC2. Table S3: The description of siderophore biosynthesis related gene cluster from *Chryseobacterium* sp. KMC2. Table S4: The description of lanthipeptide I biosynthesis related gene cluster from *Chryseobacterium* sp. KMC2. Table S5: The description of lanthipeptide II biosynthesis related gene cluster from *Chryseobacterium* sp. KMC2. Table S6: The description of NRPS-like biosynthesis related gene cluster from *Chryseobacterium* sp. KMC2. Table S7: The description of genes and metabolic pathways related to keratin utilization in *Chryseobacterium* sp. KMC2 genome.
